# Association of Seafood Consumption and Mercury Exposure With Cardiovascular and All-Cause Mortality Among US Adults

**DOI:** 10.1001/jamanetworkopen.2021.36367

**Published:** 2021-11-29

**Authors:** Yangbo Sun, Buyun Liu, Shuang Rong, Jing Zhang, Yang Du, Guifeng Xu, Linda G. Snetselaar, Robert B. Wallace, Hans-Joachim Lehmler, Wei Bao

**Affiliations:** 1 Department of Preventive Medicine, College of Medicine, The University of Tennessee Health Science Center, Memphis; 2 Department of Epidemiology, College of Public Health, University of Iowa, Iowa City; 3 Department of Nutrition and Food Hygiene, School of Public Health, Medical College, Wuhan University of Science and Technology, Wuhan, China; 4 Department of Environmental Health, School of Public Health, Jining Medical University, Jining, China; 5 Department of Occupational and Environmental Health, College of Public Health, University of Iowa, Iowa City; 6 Obesity Research and Education Initiative, University of Iowa, Iowa City; 7 Fraternal Order of Eagles Diabetes Research Center, University of Iowa, Iowa City; 8 Now with Division of Life Sciences and Medicine, University of Science and Technology of China, Hefei, China

## Abstract

**Importance:**

Although seafood is known to contain heart-healthy omega-3 fatty acids, many people choose to limit their seafood consumption because of fear of mercury exposure from seafood. It is imperative to clarify the potential health effects of current mercury exposure in contemporary populations.

**Objective:**

To examine the association of seafood consumption and mercury exposure with all-cause and cardiovascular disease (CVD)–related mortality in the US general population.

**Design, Setting, and Participants:**

This prospective cohort study included adults 20 years or older who participated in the 2003 to 2012 cycles of the National Health and Nutrition Examination Survey; data were linked to mortality records through December 31, 2015. Data analysis was performed from January to March 10, 2021.

**Exposures:**

Seafood consumption was assessed through two 24-hour dietary recalls, and mercury exposure was assessed by blood mercury levels.

**Main Outcomes and Measures:**

All-cause and CVD-related mortality. Multivariable Cox proportional hazards regression was used to estimate hazard ratios (HRs) and 95% CIs of mortality associated with usual seafood consumption and blood mercury concentration quartiles.

**Results:**

This study included 17 294 participants (mean [SD] age, 45.9 [17.1] years; 9217 [53.3%] female) with a mean (SD) blood mercury concentration of 1.62 (2.46) μg/L. During 131 276 person-years of follow-up, 1076 deaths occurred, including 181 deaths from CVD. The multivariable-adjusted HR for an increase in seafood consumption of 1 oz equivalent per day and all-cause mortality was 0.84 (95% CI, 0.66-1.07) and for CVD-related mortality was 0.89 (95% CI, 0.54-1.47). Blood mercury level was not associated with all-cause or CVD-related mortality. Comparing the highest with the lowest quartile of blood mercury concentration, the multivariable-adjusted HRs were 0.82 (95% CI, 0.66-1.05) for all-cause mortality and 0.90 (95% CI, 0.53-1.52) for CVD-related mortality.

**Conclusions and Relevance:**

In this cohort study of US adults, seafood consumption and mercury exposure with the current seafood consumption level were not significantly associated with the risk of all-cause or CVD-related mortality. These findings may inform future public health guidelines regarding mercury exposure, seafood consumption, and cardiovascular health promotion.

## Key Points

**Question:**

Are seafood consumption and mercury exposure with the current seafood consumption level associated with all-cause and cardiovascular disease (CVD)–related mortality among US adults?

**Findings:**

In this cohort study of 17 294 US adults, no association was found between an increase in seafood consumption of 1 oz equivalent per day and all-cause and CVD-related mortality. In addition, blood mercury level was not associated with all-cause or CVD-related mortality.

**Meaning:**

In this cohort study, environmental mercury exposure at the currently low to moderate level and seafood consumption were not associated with risk of all-cause or CVD-related mortality.

## Teaser

This cohort study uses National Health and Nutrition Examination Survey data to assess whether seafood consumption and mercury exposure are associated with all-cause and cardiovascular disease–related mortality.

## Introduction

Cardiovascular disease (CVD) is the leading cause of death in the US and worldwide.^1^ Greater seafood consumption was shown to be associated with reduced all-cause and CVD-related mortality in previous prospective cohort studies.^2,3^ The 2015-2020 Dietary Guidelines for Americans recommend seafood consumption of at least 8 oz or 2 servings per week for adults.^4^ However, there is a concern about seafood consumption because it is a major source of mercury exposure in daily life.^5^ Although seafood is known to contain heart-healthy omega-3 fatty acids, such as eicosapentaenoic acid (EPA) and docosahexaenoic acid (DHA),^6^ many people choose to limit their seafood consumption because of fear of mercury exposure from seafood.^7^

Emerging evidence shows that exposures to environmental chemicals are involved in the development of CVD.^8^ However, the associations of environmental mercury exposure with CVD and its risk factors are controversial, possibly because of variations in levels of mercury exposure. Early studies among Northern European or Nunavik Inuit participants with moderate- to high-level exposure to mercury (eg, mean blood mercury concentration of 10.0 μg/L [to convert to nanomoles per liter, multiply by 4.985) showed that greater exposure to mercury was associated with higher CVD risk^9^ and its risk factors^10,11^ and higher risk of CVD-related and all-cause mortality.^12^ However, such findings were not confirmed in recent studies^13,14,15^ in US populations and in some European countries with low- to moderate-level mercury exposure (eg, median serum mercury concentration of 1.40 μg/L).

It has been reported that blood mercury concentrations in US women of reproductive age decreased between 1999 and 2010.^16,17^ However, current mercury exposure levels and time trends in those levels in US adults in the past 2 decades are, to our knowledge, unknown. In addition, to our knowledge, no studies have estimated the risk of mortality associated with the current mercury exposure level in the general US population. Therefore, this study evaluated the trends in blood mercury concentrations and the association of seafood consumption and blood mercury concentrations with all-cause and CVD-related mortality in a large, nationally representative sample of US adults.

## Methods

### Study Population

This cohort study used data from the National Health and Nutrition Examination Survey (NHANES), a large-scale, ongoing, nationally representative health survey of the noninstitutionalized US population. It is conducted by the National Center for Health Statistics of the Centers for Disease Control and Prevention. NHANES survey data are collected consecutively and released every 2 years; each 2-year cycle consists of approximately 10 000 participants.^18^ The data are from population-based, cross-sectional surveys about diet, nutritional status, general health, disease history, and health behaviors.^18^ Most questionnaire data are collected during in-home interviews, and health examinations and dietary interviews are performed in specially designed and equipped mobile examination centers, which travel to locations throughout the country. The surveys use multistage, probability clusters to develop a population sample that is nationally representative of the US based on age, sex, and race and ethnicity. NHANES data along with documents on the survey methods and other information are publicly available on the NHANES website.^19^ The NHANES has been approved by the National Center for Health Statistics Ethics Review Board. All participants gave written informed consent. Because of the use of deidentified data, the University of Iowa institutional review board determined that the current study was exempt from review. This study followed the Strengthening the Reporting of Observational Studies in Epidemiology (STROBE) reporting guideline.^20^

For trends in blood mercury concentrations, data from the 2003-2004, 2005-2006, 2007-2008, 2009-2010, 2011-2012, 2013-2014, and 2015-2016 cycles of the NHANES were used, and adults 20 years or older with available data on blood mercury concentrations were included in trend analyses for blood mercury concentrations (n = 38 828). We did not include the cycles before 2003-2004 because, in those previous cycles, blood mercury levels were measured only in participants aged 1 to 5 years and female participants aged 16 to 49 years.

**Figure 1.**
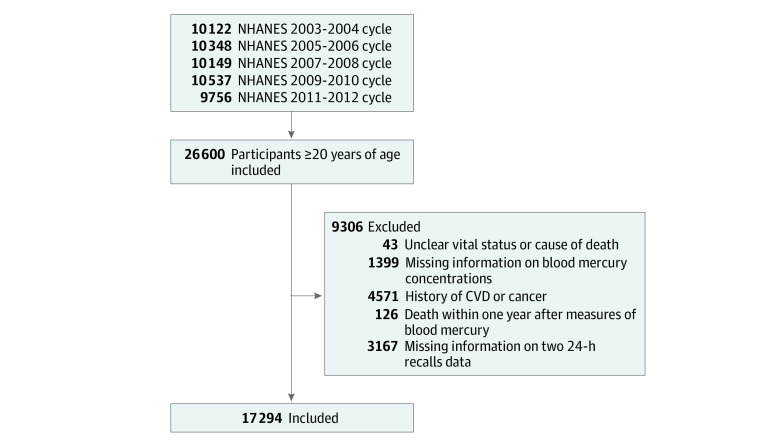
Selection of Study Participants From the 2003 to 2012 Cycles of the National Health and Nutrition Examination Survey (NHANES) CVD indicates cardiovascular disease.

For the estimation of risk of mortality associated with blood mercury concentrations and seafood consumption, data from the 2003-2004, 2005-2006, 2007-2008, 2009-2010, and 2011-2012 cycles of the NHANES were used, and 26 600 adults 20 years or older were initially included. We did not included data from the 2013-2014 cycle because blood mercury levels were examined among only one-half of the sample of participants 12 years and older in this cycle, and mortality data were available only through December 31, 2015. After the exclusion criteria were applied, 17 294 participants were included in the mortality association analysis (Figure 1).

### Assessment of Seafood Consumption

Dietary intake was assessed through two 24-hour dietary recalls. During the mobile examination center examination, a 24-hour dietary recall was administered in addition to medical and dental examinations, physiological measurements, and laboratory tests.^21^ The second dietary recall was collected by telephone approximately 3 to 10 days after the mobile examination center examination.

### Assessment of Blood Mercury Levels

Blood specimens were collected at the mobile examination centers and sent to the Division of Laboratory Sciences, National Center for Environmental Health, Centers for Disease Control and Prevention, for analysis. All laboratory data were standardized under the NHANES quality assurance and quality control process.^22^ Inductively coupled plasma mass spectrometry (PerkinElmer ELAN 6100) was used to measure mercury levels in whole blood samples in the NHANES 2003-2012 cycle, with a limit of detection of 0.14 or 0.2 μg/L for 2003-2004, 0.2 or 0.32 μg/L for 2005-2006, 0.28 μg/L for 2007-2008, 0.33 μg/L for 2009-2010, and 0.16 μg/L for 2011-2012.^23^ A conservative approach suggested by the National Center for Health Statistics is to use the highest limit of detection fill value for all years of data being analyzed.^24^ Thus, in this analysis, values below 0.33 μg/L would be considered below the limit of detection. Values below the limit of detection were imputed by using a value equal to the detection limit divided by the square root of 2.

### Assessment of Outcomes

Death status for each participant was determined using the NHANES Public Use Linked Mortality File, which was created to permit long-term follow-up study of participants in the NHANES.^25^ This file contains information based on the results of a probabilistic match between the NHANES and the National Death Index records to ascertain the vital status of each eligible NHANES participant through December 31, 2015. Underlying causes of death were classified according to the codes of the *International Statistical Classification of Diseases and Related Health Problems, Tenth Revision (ICD-10)* for deaths occurring between 1999 and 2015.^26^ Deaths from CVD were identified according to *ICD-10* codes I00 to I78. Persons who survived were administratively censored on December 31, 2015. Follow-up time for each person was calculated as the difference between the NHANES examination date and the last known date alive or censored from the NHANES mortality file.

### Covariates

Information on age; sex; race and ethnicity; family income; educational level; smoking status; alcohol intake; physical activity; dietary intake; history of diabetes, hypertension, CVD, and cancer; and family history of CVD was ascertained by standardized questionnaires. Race and ethnicity were categorized as non-Hispanic White, non-Hispanic Black, Mexican American, and other racial or ethnic group (other Hispanic, other races, and multiracial). The family income-to-poverty ratio was categorized as less than 1.0, 1.0 to 1.9, 2.0 to 3.9, and 4.0 or higher.^27^ A higher income-to-poverty ratio represents a higher family income status. Educational level was categorized as less than high school, high school, and college or above. Participants were categorized as nonsmokers, past smokers, and current smokers based on their responses to questions about smoking at least 100 cigarettes during their lifetime and whether they were currently smoking. Overall diet quality was indicated by the Healthy Eating Index–2010, which is scored on the basis of the intake levels of 12 dietary components, including total fruit, whole fruit, total vegetables, greens and beans, whole grains, dairy, total protein foods, seafood and plant proteins, fatty acids, refined grains, sodium, and empty calories (ie, energy from solid fats, alcohol, and added sugars).^28^ Then, the simple Healthy Eating Index–2010 scoring algorithm (per person) was used to calculate the individual score.^29^ Consumption of whole grains, total fruits, total vegetables, red meat, and poultry was expressed as the mean intake of each food from two 24-hour recalls. Total energy intake, alcohol intake, EPA and DHA intake, and selenium intake were calculated using the US Department of Agriculture’s Food and Nutrient Database for Dietary Studies^28,30^ and was expressed as the mean intake of each food or nutrient from two 24-hour recalls. Current alcohol intake was categorized as none (0 g/d), moderate drinking (0.1-27.9 g/d for men and 0.1-13.9 g/d for women), and heavy drinking (≥28 g/d for men and ≥14 g/d for women). Physical activity was classified as follows into 3 groups according to standards appropriate for each cycle: (1) below (<600 metabolic equivalent [MET] min/wk or 150-minute/wk of moderate-intensity exercise), (2) meet (600-1200 MET min/wk or 150-300-minute/wk of moderate-intensity exercise), or (3) exceed (>1200 MET min/wk or 300-minute/wk of moderate-intensity exercise).^31^ Trained technicians measured weight and height. Body mass index (BMI) was calculated as weight in kilograms divided by height in meters squared and grouped as less than 25, 25 to 29.9, and 30 or greater. Serum cholesterol levels were measured according to standard methods and were categorized into 3 groups (<200, 200-239, and ≥240 mg/dL [to convert to millimoles per liter, multiply by 0.0259]).

### Statistical Analysis

Data analysis was performed from January to March 10, 2021. All statistical analyses accounted for the complex, multistage, stratified, cluster-sampling design of the NHANES by using sample weights, strata, and primary sampling units embedded in the NHANES data.^32^ Comparisons of characteristics across blood mercury concentration quartiles were performed using linear regression for continuous variables and χ^2^ tests for categorical variables.

The association between usual seafood intake and mortality was estimated by applying the National Cancer Institute method using data from the two 24-hour recalls^33^ with the MIXTRAN and INDIVINT macros in SAS, version 2.1 (SAS Institute, Inc). To estimate the difference in risk for mortality between individuals with a mean of no consumption and 1.0 oz of fish per day, the calculations involved fitting a nonlinear mixed model using the MIXTRAN macro with weights and with age, sex, race and ethnicity, educational level, and income level as covariates. After using the results of this fit as input for the INDIVINT macro, the INDIVINT macro results were used in a multivariable Cox proportional hazards regression model to estimate hazard ratios (HRs) and 95% CIs of mortality associated with usual seafood consumption and blood mercury concentration quartiles. For mortality associated with seafood consumption, model 1 adjusted for age, sex, and race and ethnicity. Model 2 further adjusted for educational level, family income-to-poverty ratio, smoking status, alcohol intake, physical activity, total energy intake, and consumption of whole grains, total fruits, total vegetables, red meat, and poultry. Model 3 further adjusted for BMI, history of diabetes, history of hypertension, family history of CVD, and total cholesterol level. For mortality associated with blood mercury concentration quartiles, model 1 adjusted for age, sex, and race and ethnicity. Model 2 further adjusted for educational level, family income-to-poverty ratio, smoking status, alcohol intake, physical activity, total energy intake, overall diet quality indicated by Healthy Eating Index–2010 score, dietary EPA and DHA intake, blood cadmium and lead levels, and dietary selenium intake (because the trace element selenium showed protection against mercury toxicity in some experimental models).^5^ Model 3 further adjusted for BMI, history of diabetes, history of hypertension, family history of CVD, and total cholesterol level. All analyses were performed using survey procedures in SAS, version 9.4.^34^ All hypothesis tests were 2-sided. *P* < .05 was considered statistically significant.

## Results

A total of 17 294 participants (mean [SD] age, 45.9 [17.1] years; 9217 [53.3%] female) with a mean (SD) blood mercury concentration of 1.62 (2.46) μg/L were included in the association analysis. During 131 276 person-years of follow-up, 1076 deaths occurred, including 181 deaths from CVD. Blood mercury concentrations correlated weakly with fish consumption (*r* = 0.23, *P* < .001), dietary intake of EPA and DHA (*r* = 0.21, *P* < .001), and dietary selenium intake (*r* = 0.12, *P* < .001). Participants with higher blood mercury concentrations were more likely to be older; have higher educational levels and family incomes; be current smokers; have higher alcohol intake and physical activity levels; have higher diet quality; have higher intake of EPA, DHA, and selenium; have lower BMI; have lower blood cadmium and higher blood lead levels; and be less likely to have diabetes and a family history of CVD at baseline, although they had higher total cholesterol levels (Table 1).

**Table 1.** Characteristics of the Study Population in the Mortality Association Analysis^a^

Characteristic	Blood mercury quartile				
	Quartile 1 (≤0.48 μg/L) (n = 4294)	Quartile 2 (0.49-0.89 μg/L) (n = 4279)	Quartile 3 (0.90-1.78 μg/L) (n = 4486)	Quartile 4 (≥1.79 μg/L) (n = 4235)	*P* value^b^
Age, mean (SE), y	41.5 (0.4)	43.6 (0.4)	45.3 (0.3)	46.6 (0.4)	<.001
Sex					
Female	53.3 (0.9)	53.6 (0.9)	53.8 (0.9)	50.4 (0.9)	.03
Male	46.7 (0.9)	46.4 (0.9)	46.2 (0.9)	49.6 (0.9)	
Race and ethnicity					
Mexican American	11.5 (1.3)	11.5 (1.2)	8.2 (0.8)	4.2 (0.4)	<.001
Non-Hispanic					
Black	8.9 (1.0)	11.4 (1.0)	12.9 (1.0)	.4 (0.9)	
White	72.7 (2.1)	68.3 (1.8)	69.0 (1.8)	68.3 (1.8)	
Other^c^	6.9 (0.6)	8.7 (0.7)	9.8 (0.8)	17.1 (1.2)	
Educational level					
Less than high school	23.2 (1.2)	17.7 (0.8)	14.7 (0.9)	10.3 (0.6)	<.001
High school	28.0 (1.0)	26.5 (1.0)	22.8 (0.8)	17.5 (0.8)	
College or above	48.8 (1.6)	55.8 (1.3)	62.5 (1.1)	72.2 (1.0)	
Family income-to-poverty ratio					
<1.0	17.9 (1.1)	14.6 (0.8)	11.1 (0.7)	6.9 (0.5)	<.001
1.0-1.9	24.3 (1.0)	20.3 (0.9)	16.8 (0.7)	13.2 (0.7)	
2.0-3.9	29.3 (1.1)	30.2 (0.9)	29.4 (1.0)	25.1 (1.1)	
≥4.0	22.9 (1.2)	29.4 (1.1)	37.7 (1.4)	49.9 (1.4)	
Missing	5.2 (0.5)	5.5 (0.4)	5.1 (0.5)	5.0 (0.5)	
Smoking status					
Nonsmoker	53.2 (1.4)	54.5 (0.9)	57.3 (1.0)	56.7 (1.2)	<.001
Current	18.7 (1.0)	21.6 (0.8)	23.1 (0.9)	27.0 (1.0)	
Ever	28.0 (1.1)	23.8 (0.9)	19.5 (0.7)	16.3 (0.9)	
Alcohol intake^d^					
Nondrinker	74.5 (1.1)	68.9 (1.2)	62.9 (1.0)	50.8 (1.2)	<.001
Moderate	12.9 (0.9)	16.7 (0.7)	20.6 (0.9)	26.5 (0.9)	
Heavy	12.6 (0.9)	14.4 (0.9)	16.5 (0.8)	22.8 (1.0)	
Physical activity, MET min/wk					
<600	41.1 (1.1)	37.8 (1.1)	35.1 (1.1)	29.6 (1.0)	<.001
≥600-1199	12.4 (0.7)	12.5 (0.7)	14.7 (0.8)	14.1 (0.7)	
≥1200	46.5 (1.2)	49.7 (1.2)	50.2 (1.1)	56.3 (1.1)	
Total energy intake, mean (SE), kcal/d	2170 (19)	2179 (21)	2168 (16)	2156 (18)	.87
HEI-2010 score, mean (SE)	44.7 (0.4)	47.4 (0.3)	50.0 (0.3)	54.3 (0.4)	<.001
EPA and DHA intake, mean (SE), mg/d	51.3 (2.5)	82.5 (3.8)	120.0 (5.6)	202.4 (7.7)	<.001
Selenium intake, mean (SE), μg/d	106.1 (1.0)	111.5 (1.1)	114.1 (1.0)	123.5 (1.2)	<.001
BMI, mean (SE)	29.1 (0.2)	29.1 (0.2)	28.7 (0.1)	27.5 (0.1)	<.001
History of diabetes	7.1 (0.5)	7.1 (0.6)	5.7 (0.4)	4.9 (0.3)	.03
History of hypertension	38.4 (1.0)	39.8 (1.2)	42.0 (1.0)	40.3 (1.1)	.06
Family history of CVD	13.4 (0.7)	13.0 (0.8)	12.6 (0.6)	10.8 (0.7)	.04
Total cholesterol level, mean (SE), mg/dL	194.4 (0.9)	197.8 (0.6)	200.2 (0.7)	202.9 (1.0)	<.001
Blood cadmium level, mean (SE), μg/L	0.57 (0.02)	0.49 (0.01)	0.49 (0.01)	0.47 (0.01)	<.001
Blood lead level, mean (SE), μg/dL	1.51 (0.05)	1.53 (0.04)	1.55 (0.03)	1.69 (0.03)	<.001

Abbreviations: BMI, body mass index (calculated as weight in kilograms divided by height in meters squared); CVD, cardiovascular disease; DHA, docosahexaenoic acid; EPA, eicosapentaenoic acid; HEI, Healthy Eating Index–2010; MET, metabolic equivalent.

SI conversion factors: To convert cadmium to nanomoles per liter, multiply by 8.89; cholesterol to millimoles per liter, multiply by 0.0259; lead to micromoles per liter, multiply by 0.0483; and mercury to nanomoles per liter, multiply by 4.985.

^a^ Data are presented as weighted percentage of participants (SE) unless otherwise indicated.

^b^ *P* < .05 was considered statistically significant.

^c^ Other includes other Hispanic, other races, and multiracial.

^d^ Nondrinker was defined as 0 g/d; moderate drinking, 0.1 to 28 g/d for men and 0.1 to 14 g/d for women; and heavy drinking, 28 g/d or more for men and 14 g/d or more for women.

### Mortality Associated With Usual Seafood Intake

The association between usual seafood intake and mortality is shown in Table 2. After adjustment for age, sex, race and ethnicity, socioeconomic status, dietary and lifestyle factors, BMI, history of diabetes and hypertension, family history of CVD, and total cholesterol levels, the multivariable-adjusted HRs for an increase in seafood consumption of 1 oz equivalent per day and all-cause mortality was 0.84 (95% CI, 0.66-1.07) and for CVD-related mortality was 0.89 (95% CI, 0.54-1.47), indicating no association between an increase in seafood consumption of 1 oz equivalent per day and all-cause and CVD-related mortality.

**Table 2.** Association of Usual Seafood Intake With All-Cause and CVD-Related Mortality Among 17 294 Participants From the 2003 to 2012 Cycles of the National Health and Nutrition Examination Survey

Mortality	HR per 1 oz equivalent per day increase (95% CI)
All cause	
Model 1^a^	0.60 (0.47-0.77)^b^
Model 2^c^	0.84 (0.66-1.06)
Model 3^d^	0.84 (0.66-1.07)
CVD related	
Model 1^a^	0.54 (0.30-0.98)^b^
Model 2^c^	0.87 (0.52-1.47)
Model 3^d^	0.89 (0.54-1.47)

Abbreviations: CVD, cardiovascular disease; HR, hazard ratio.

^a^ Model 1 was adjusted for age, sex, and race and ethnicity.

^b^ Statistically significant.

^c^ Model 2 was adjusted for the variables in model 1 plus educational level, family income-to-poverty ratio, smoking status, alcohol intake, physical activity, total energy intake, and consumption of whole grains, total fruits, total vegetables, red meat, and poultry.

^d^ Model 3 was adjusted for the variables in model 2 plus body mass index, history of diabetes, history of hypertension, family history of CVD, and total cholesterol levels.

### Trends in Blood Mercury Concentrations in US Adults From 2003 to 2016

**Figure 2.**
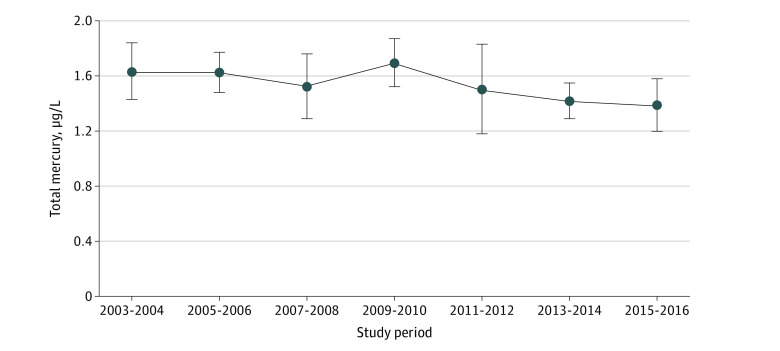
Trends in Total Blood Mercury Concentrations Among US Adults From the 2003 to 2016 Cycles of the National Health and Nutrition Examination Survey Error bars indicate 95% CIs. To convert total mercury to nanomoles per liter, multiply by 4.99.

During 2003 to 2016, mean (SE) blood mercury concentrations remined generally unchanged from 2003-2004 (1.63 [0.09] μg/L) to 2009-2010 (1.70 [0.08] μg/L) (*P* = .61). There was a mean (SE) downward trend from 2009-2010 (1.70 [0.08] μg/L) to 2015-2016 (1.39 [0.09] μg/L) (*P* < .001) (Figure 2).

### Mortality Associated With Blood Mercury Concentrations

Blood mercury concentrations were not associated with risk of all-cause or CVD-related mortality (Table 3). After adjustment for age, sex, race and ethnicity, socioeconomic status, dietary and lifestyle factors, BMI, history of diabetes and hypertension, family history of CVD, and total cholesterol levels, the multivariable-adjusted HRs for all-cause mortality were 1.00 (reference) for blood mercury quartile 1 (≤0.48 μg/L), 1.00 (95% CI, 0.83-1.21) for blood mercury quartile 2 (0.49-0.89 μg/L), 0.81 (95% CI, 0.65-1.02) for blood mercury quartile 3 (0.90-1.78 μg/L), and 0.84 (95% CI, 0.67-1.05) for blood mercury quartile 4 (≥1.79 μg/L) (*P* = .11 for trend). The multivariable-adjusted HRs for CVD mortality were 1.00 (reference) for blood mercury quartile 1 (≤0.48 μg/L), 1.23 (95% CI, 0.78-1.93) for blood mercury quartile 2 (0.49-0.89 μg/L), 1.24 (95% CI, 0.79-1.96) for blood mercury quartile 3 (0.90-1.78 μg/L), and 0.88 (95% CI, 0.51-1.50) for blood mercury quartile 4 (≥1.79 μg/L) (*P* = .40 for trend).

**Table 3.** Association of Blood Mercury Concentrations With All-Cause and CVD-Related Mortality Among 17 294 Participants From the 2003 to 2012 Cycles of the National Health and Nutrition Examination Survey

Mortality	HR (95% CI), by blood mercury quartile				*P* value for trend^a^
	Quartile 1 (≤0.48 μg/L)	Quartile 2 (0.49-0.89 μg/L)	Quartile 3 (0.90-1.78 μg/L)	Quartile 4 (≥1.79 μg/L)	
All cause					
Model 1^b^	1 [Reference]	0.84 (0.70-1.02)	0.60 (0.49-0.74)^c^	0.53 (0.43-0.66)^c^	<.001
Model 2^d^	1 [Reference]	0.98 (0.81-1.20)	0.79 (0.64-0.99)^c^	0.81 (0.65-1.01)	.06
Model 3^e^	1 [Reference]	1.00 (0.83-1.21)	0.81 (0.65-1.02)	0.84 (0.67-1.05)	.11
CVD related					
Model 1^b^	1 [Reference]	1.07 (0.69-1.68)	0.89 (0.57-1.40)	0.53 (0.33-0.85)^c^	.003
Model 2^d^	1 [Reference]	1.24 (0.79-1.97)	1.20 (0.75-1.90)	0.85 (0.50-1.44)	.32
Model 3^e^	1 [Reference]	1.23 (0.78-1.93)	1.24 (0.79-1.96)	0.88 (0.51-1.50)	.40

Abbreviations: CVD, cardiovascular disease; HR, hazard ratio.

SI conversion factor: To convert mercury to nanomoles per liter, multiply by 4.985.

^a^ *P* < .05 was considered statistically significant.

^b^ Model 1 was adjusted for age, sex, and race and ethnicity.

^c^ Statistically significant.

^d^ Model 2 was adjusted for the variables in model 1 plus educational level, family income-to-poverty ratio, smoking status, alcohol intake, physical activity, total energy intake, and consumption of whole grains, total fruits, total vegetables, red meat, and poultry.

^e^ Model 3 was adjusted for the variables in model 2 plus body mass index, history of diabetes, history of hypertension, family history of CVD, and total cholesterol levels.

## Discussion

In this large, nationally representative population, usual seafood consumption was not associated with the risk of mortality. Consistent with a previous report from 2011 to 2016,^35^ the current mercury exposure level in US adults was similar to the low level previously reported in central Europe but much lower than that in other European countries that have high fish consumption^36^; the low to moderate level was steady during the past 10 years. In addition, at the current low to moderate level of mercury exposure, higher blood mercury concentrations were not associated with risk of all-cause or CVD-related mortality among US adults after adjustment for demographic, socioeconomic, dietary, and lifestyle factors; health status; and family history of CVD. Moreover, the lack of association between blood mercury concentrations and mortality was independent of dietary EPA and DHA intake or selenium intake. These findings do not support an association of usual levels of concentrations to environmental mercury with mortality among US adults.

This study provides important evidence regarding the associations between the current low to moderate level of mercury exposure with the current seafood consumption and mortality in the general population. Because mercury exposure levels could be different among settings, it is important and reasonable to consider how mercury exposure levels in this study and their associations with mortality compare with those in previous studies with similar mercury exposure levels. The result that blood mercury level was not associated with all-cause mortality was consistent with a study^13^ that found that serum mercury level was not associated with all-cause mortality in Swedish women with a low to moderate level of exposure to mercury (median serum mercury concentration, 1.40 μg/L). Consistent with findings regarding CVD-related mortality in the current study, a previous study^14^ using data from the US Health Professionals Follow-up Study and Nurses’ Health Study found no significant association between mercury exposure (mean toenail mercury concentration, 0.25 μg/g) and risk of CVD. Similar results of mercury levels and CVD incidence were reported in a previous study^15^ in Spain (mean toenail mercury concentration, 0.66 μg/g) in which participants had a similarly low to moderate level of mercury. However, these results were inconsistent with 2 smaller studies in which hair or toenail mercury concentrations were positively associated with all-cause mortality, CVD-related mortality,^37^ or myocardial infarction.^38^ The smaller study^38^ (N = 1408) in Europe was restricted to male patients with nonfatal myocardial faction who survived until hospitalization; thus, selection bias was possible. The larger study^37^ (N = 1871) in men from Finland found a positive association with all-cause and CVD-related mortality but without a dose-response association. This finding could possibly be explained by the lower serum selenium concentrations among Finnish participants because a previous experimental study^5^ found that the trace element selenium provides cardiovascular protection against the toxic effects of mercury. Nevertheless, the generalizability of these studies was limited because they included only men or largely included White adults and adults with high educational levels.

The associations between seafood consumption and health were inconsistent in previous studies.^2,6,39,40,41,42^ Several previous studies reported a significant association of seafood consumption with lower risk of all-cause and CVD-related mortality,^2,39^ likely because of the beneficial effects of EPA and DHA in fish.^6^ However, consistent with our findings, other studies^40,41^ found that seafood consumption was not associated with CVD-related mortality. Mercury in blood generally reflects recent exposure to methylmercury (mainly via fish consumption).^42^ In the current study, higher blood mercury concentrations were not associated with lower all-cause and CVD-related mortality. To our knowledge, there is no biological explanation for cardiovascular and mortality benefits of methylmercury, although in an animal experimental study,^43^ a low dose of methylmercury increased the carotid artery diameter, which reduced peripheral resistance and decreased blood pressure. Therefore, the observed associations between mercury and mortality could be attributable to confounding by other factors associated with mercury that lower the risk of death. For example, blood mercury level was associated with higher seafood consumption and factors associated with higher seafood consumption, including higher physical activity levels, higher EPA and DHA intake, and higher selenium intake. However, adjustment for dietary EPA and DHA intake in this study did not alter the associations. Furthermore, the associations were similar when the analysis was restricted to participants with seafood consumption less than 2 times per week. Nevertheless, residual confounding was still possible because of imperfect measurement of these factors or other unmeasured factors. Another explanation for the null associations between blood mercury concentrations and mortality may be the possible cardiovascular protection by the trace element selenium.^5^ However, the adjustment for dietary selenium intake did not alter the findings. Finally, although total mercury concentrations in NHANES reflect primarily methylmercury, elemental or inorganic mercury in blood may also have contributed to the total mercury exposures, mainly via dental amalgams, environment, or drug use.^44^ Animal studies^45,46,47^ have found that inorganic mercury could decrease blood pressure. However, the cardiovascular effects of inorganic mercury in humans are unclear. In addition, in NHANES, blood inorganic mercury in participants with higher total blood mercury concentrations may constitute a smaller proportion of total blood mercury than it does at lower concentrations.^48^

The findings that the current low to moderate level of exposure to environmental mercury in US adults not being associated with risk of all-cause or CVD-related mortality does not support the adverse effects of low to moderate level of mercury exposure for mortality. At the current mercury exposure levels in US adults, this study does not suggest a need to change the current dietary guidelines that recommend seafood consumption as part of a healthy diet for US adults in terms of concerns about the cardiovascular effects of mercury. It is noteworthy that these findings were not relevant to the dietary guidelines for specific subpopulations, such as pregnant women, for whom attention to the neurotoxic effects of methylmercury exposure from specific fish species on their children is important.^4^

### Strengths and Limitations

This study has strengths. The study included a nationally representative sample, which facilitates the generalization of the findings to the US general population. In addition, with the detailed and high-quality data collection in the NHANES, this study was able to control potential confounding effects from a variety of demographic, socioeconomic, dietary, and lifestyle factors; health status; and family history of CVD. For seafood consumption, the National Cancer Institute method was used to estimate the usual intake based on information from two 24-hour recalls, which may have reduced measurement errors.^33^

This study also has limitations. First, only 1 measure of blood mercury concentration was available; thus, it may not indicate long-term exposure, which may have contributed to the null association. Second, although the blood total mercury concentrations reflected primarily methylmercury,^49^ inorganic mercury may still contribute to the associations of total blood mercury concentrations with mortality. It was not possible to distinguish their separate effects, although as previously mentioned, inorganic mercury may constitute a smaller proportion of blood total mercury at higher concentrations than it does at lower concentrations.^48^ Third, dietary EPA and DHA intake and selenium intake were assessed through two 24-hour dietary recalls, which may have been subject to misclassification. Given that the associations of mercury and mortality may have been partly confounded by the beneficial effects of seafood consumption (mainly via EPA and DHA) and selenium intake, misclassification of dietary EPA and DHA intake or dietary selenium intake might account for the null association. Fourth, the number of deaths from CVD in this study was small, which may have limited the statistical power to detect a significant association. Fifth, despite adjustment for a variety of potential confounders, residual confounding may still exist.

## Conclusions

In this cohort study of a national representative population of US adults, environmental mercury exposure at the currently low to moderate level was not associated with risk of all-cause or CVD-related mortality. This result was independent of dietary EPA and DHA intake or selenium intake. These findings may inform future public health guidelines regarding mercury exposure, seafood consumption, and cardiovascular health promotion.
